# Timing and Type of Alcohol Consumption and the Metabolic Syndrome - ELSA-Brasil

**DOI:** 10.1371/journal.pone.0163044

**Published:** 2016-09-19

**Authors:** Bruna Angelo Vieira, Vivian Cristine Luft, Maria Inês Schmidt, Lloyd Ellwood Chambless, Dora Chor, Sandhi Maria Barreto, Bruce Bartholow Duncan

**Affiliations:** 1 Graduate Studies Program in Epidemiology and Hospital de Clínicas de Porto Alegre, Federal University of Rio Grande do Sul (UFRGS), Porto Alegre, Brazil; 2 Food and Nutrition Research Center, Hospital de Clínicas de Porto Alegre (HCPA-UFRGS), Porto Alegre, Brazil; 3 Department of Biostatistics, University of North Carolina, Chapel Hill, North Carolina, United States of America; 4 Department of Epidemiology, National School of Public Health, Oswaldo Cruz Foundation, Rio de Janeiro, Brazil; 5 Graduate Studies Program of Public Health, Federal University of Minas Gerais (UFMG), Belo Horizonte, Brazil; National Institue on Drug Abuse, UNITED STATES

## Abstract

The prevalence of the metabolic syndrome is rising worldwide. Its association with alcohol intake, a major lifestyle factor, is unclear, particularly with respect to the influence of drinking with as opposed to outside of meals. We investigated the associations of different aspects of alcohol consumption with the metabolic syndrome and its components. In cross-sectional analyses of 14,375 active or retired civil servants (aged 35–74 years) participating in the Brazilian Longitudinal Study of Adult Health (ELSA-Brasil), we fitted logistic regression models to investigate interactions between the quantity of alcohol, the timing of its consumption with respect to meals, and the predominant beverage type in the association of alcohol consumption with the metabolic syndrome. In analyses adjusted for age, sex, educational level, income, socioeconomic status, ethnicity, smoking, body mass index, and physical activity, light consumption of alcoholic beverages with meals was inversely associated with the metabolic syndrome (≤4 drinks/week: OR = 0.85, 95%CI 0.74–0.97; 4 to 7 drinks/week: OR = 0.75, 95%CI 0.61–0.92), compared to abstention/occasional drinking. On the other hand, greater consumption of alcohol consumed outside of meals was significantly associated with the metabolic syndrome (7 to 14 drinks/week: OR = 1.32, 95%CI 1.11–1.57; ≥14 drinks/week: OR = 1.60, 95%CI 1.29–1.98). Drinking predominantly wine, which occurred mostly with meals, was significantly related to a lower syndrome prevalence; drinking predominantly beer, most notably when outside of meals and in larger quantity, was frequently associated with a greater prevalence. In conclusion, the alcohol—metabolic syndrome association differs markedly depending on the relationship of intake to meals. Beverage preference—wine or beer—appears to underlie at least part of this difference. Notably, most alcohol was consumed in metabolically unfavorable type and timing. If further investigations extend these findings to clinically relevant endpoints, public policies should recommend that alcohol, when taken, should be preferably consumed with meals.

## Introduction

The metabolic syndrome is a complex of interrelated risk factors which predict cardiovascular disease and type 2 diabetes [1]. Its prevalence is rising worldwide, representing an important public health problem [2–4]. It is related to many lifestyle behaviors of modern world [5], including alcohol consumption, a common exposure [6,7].

Moderate alcohol intake appears to offer benefits for health, including a lower risk of cardiovascular disease [8,9], diabetes [10] and mortality [8–12]. However, few epidemiological studies have evaluated the role of the timing of alcohol consumption, i.e., with or outside of meals, in these associations. Some smaller studies have found more favorable associations of alcohol consumption with metabolic markers [7,13], general mortality [14] and cardiovascular events when it was taken with meals [15]. However, to our knowledge, none have to date investigated the impact of the timing of consumption with respect to the metabolic syndrome.

Considering that investigation of alcohol´s relationship to the metabolic syndrome [16–19] and different syndrome components [20–27] has not considered the moment of ingestion, we propose to investigate the extent to which the timing of alcohol consumption and beverage type alter these associations.

## Materials and Methods

The Brazilian Longitudinal Study of Adult Health (ELSA-Brasil), as previously described [28,29], is a prospective cohort study designed to investigate principally diabetes and cardiovascular diseases. Study volunteers, enrolled in six Brazilian states, are active or retired civil servants (35–74 years) of universities or research institutions [28]. Of a total of 15,105 participants, we excluded from these baseline cross-sectional analyses individuals with incomplete alcohol consumption data (N = 25); not having the data required for metabolic syndrome classification (N = 288) or for covariates used in modeling (N = 417), leaving 14,375 subjects for analysis. ELSA-Brasil was approved by the Ethics Committees of the Hospital de Clínicas de Porto Alegre (06–194), Hospital Universitário da Universidade de São Paulo (669/06), Fundação Oswaldo Cruz (343/06), Universidade Federal de Minas Gerais (186/06), Universidade Federal da Bahia (027–06) and Universidade Federal do Espírito Santo (041/06). All participants gave written consent to participate.

Participants were instructed not to consume alcoholic beverages on the day before the examination and to fast for 12 hours. We obtained sociodemographic data and habits of alcohol consumption and physical activity in leisure-time by interview [30,31]. Participants replied to a standard questionnaire on frequency and quantity consumed of each type of alcohol beverage (drinks/week). We considered as one drink of alcohol 1 can/bottle of beer (350ml), 1 glass of wine (120–150 ml) or 1 shot of spirits (40 ml). Those abstaining or consuming less than 1 drink/week were considered not to drink regularly, and those consuming at least 1 drink/week were grouped into four categories (up to 4 drinks/week, 4 to 7 drinks/week, 7 to 14 drinks/week, more than 14 drinks/week). To characterize the timing of alcohol consumption, we asked “Considering all the alcoholic beverages that you consume, how often do you drink with meals?”, and analyzed responses as: “Most frequently with meals”, “Both with and outside of meals” and “Most frequently outside of meals”. Binge drinking was defined as consuming 5 or more drinks within a two hour period more than once a month. Wine (or beer) was considered the predominant beverage if it constituted >50% of total consumption. Leisure-time physical activity was defined by the weighted sum of the time of physical activity per week, using the long version of the International Physical Activity Questionnaire (IPAQ), according to its guidelines for data processing and analysis. Metabolic equivalent minutes (MET-minutes) were computed for walking, moderate-intensity activities, and vigorous-intensity activities using the following formulas: Walking MET-minutes/week = 3.3 * walking minutes * walking ‘days’; Moderate MET-minutes/week = 4.0 * moderate-intensity activity minutes * moderate days; Vigorous MET-minutes/week = 8.0 * vigorous-intensity activity minutes * vigorous-intensity days. A combined total physical activity MET-minutes/week was computed as the sum of Walking + Moderate + Vigorous METs/week scores [32]. Ethnicity was defined by the participant’s self-declared skin color/race following the standard approach used to obtain official Brazilian statistics.

The definition of social class was derived from the ELSA database and takes into consideration a detailed analysis of participants' current job tasks. First, the occupation of each participant was classified using the Brazilian job-occupational matrix. Next, the expected income of each participant was derived from his educational level (average market value), and his socioeconomic status was estimated using the mean of his observed and expected income. Then, the average of the means of participants having the same occupation was used to obtain an occupational socioeconomic status score. Using these occupational socioeconomic status scores, occupations were grouped into social classes so as to achieve a minimum intra-stratum and maximum between-stratum variance of the scores. Finally, for this report, social class was categorized into three levels: high, middle, and low.

We measured weight, waist circumference and height while fasting, and defined nutritional status using the standard Body Mass Index (BMI) categories. We used the average of the last two of three blood pressure measurements in analyses [33].

Blood glucose was determined by the enzymatic hexokinase method; and total cholesterol, triglycerides, high-density lipoprtein cholesterol (HDL-C) and low-density lipoprotein cholesterol (LDL-C) by enzymatic calorimetric methods [34] at a central laboratory.

The metabolic syndrome was defined as the presence of at least three of the following factors: elevated fasting glucose (≥100 mg/dL or use of hypoglycemic medication), elevated triglycerides (≥150 mg/dL or use of fibrates and/or nicotinic acid), low HDL-C (<40 mg/dL for men, <50 mg/dL for women, or use of fibrates and/or nicotinic acid), elevated blood pressure (systolic blood pressure ≥135 mmHg and/or diastolic blood pressure ≥85 mmHg or confirmed use of antihypertensive medication) and abdominal obesity (waist circumference ≥ 94 cm for men and ≥ 80 cm for women) [1].

We present descriptive statistics for continuous variables as median (interquartile range) and for categorical data as absolute and relative frequencies. We investigated the association of aspects of alcohol consumption with the presence of metabolic syndrome and its components (as dichotomous outcomes) through construction of adjusted logistic regression models using a parameterization to represent interactions between the amount of alcohol consumed, the predominant beverage type (wine, beer or other) and the timing of consumption (mostly with meals, mostly outside of meals, or equivalently with and outside of meals).

Because of categories that were empty by design, e.g. non-drinker x predominantly wine, or non-drinker x drinks with meals, we did not model the associations in the usual way, with main effects and interactions of those main effects. Rather, we set up the model with beta coefficients for the categories of quantity consumed for each time/type, where each beta coefficient represented a difference between non-drinkers and drinkers at a particular quantity of alcohol consumed. To test the global association of the metabolic syndrome with the quantity of alcohol consumed for a particular timing and/or type of beverage, we tested whether the beta coefficients for the categories of quantity consumed for that time/type, taken together, differed from zero (chi-square with a degree of freedom equal to the number of the quantity categories minus one). The reference category was always those who do not drink regularly. We report differences found at specific quantities ingested only if overall significance was confirmed in these initial tests.

Within this strategy, we tested the global difference in outcomes between the timings of ingestion by evaluating the significance of the difference between the sets of coefficients for ingestion with meals and outside of meals, when each is taken as a group [chi-square with degrees of freedom = 2 * (number of alcohol levels minus one)].

Analyzes were performed using SAS software (Statistical Analysis System, SAS Institute Inc., Cary, N.C.), version 9.4, and statistical significance was defined as p<0.05.

## Results

Overall, 7,835 (54.5%) participants were women, 7,514 (52.3%) white, 7,588 (52.8%) had a university degree, and 5,937 (41.3%) were classified as middle class. Most (8.208, 57.1%) had never smoked; 9,064 (63.1%) were either overweight or obese. We observed that 6,933 (48.2%) reported regular alcohol consumption (at least 1 drink/week). Of these, 3,306 (47.7%) consumed up to 4 drinks/week, 1,365 (19.7%) between 4 and 7 drinks/week, 1,436 (20.7%) between 7 and 14 drinks/week and 826 (11.9%) more than 14 drinks/week. Among those who reported drinking alcohol regularly, 3,681 (53.1%) drank alcohol most frequently outside of meals, 2,714 (39.2%) most frequently with meals and only 538 (7.8%) drank equivalently during and outside of meals. With respect to beverage type, 2,035 (29.3%) drank predominantly wine, 3,943 (56.9%) predominantly beer, 334 (4.8%) predominantly spirits, and 621 (9.0%) drank without a predominant beverage. Of those who drank mostly with meals, 1,318 (48.6%) drank predominantly wine. Of those who drank mostly outside of meals, 2,671 (72.6%) drank predominantly beer.

We ascertained the metabolic syndrome in 6,325 (44.0%) individuals. Among the entire sample, 6,380 (44.4%) individuals had high blood pressure, 10,300 (71.7%) elevated fasting glucose, 4,505 (31.3%) elevated triglycerides, 2,647 (18.4%) reduced HDL-C and 9,037 (62.9%) elevated waist circumference.

Table 1 presents sociodemographic, anthropometric, metabolic and behavioral characteristics of the study population by different quantities of alcohol consumed. In general, all measured characteristics were statistically associated (p<0.001) with the quantity of alcohol consumed. Among individuals who consumed larger quantities, more were men, smokers, and overweight. As the amount consumed per week increased, the proportion of subjects consuming predominantly beer increased as well.

**Table 1:** Characteristics of participants, by quantity of alcohol consumed. ELSA-Brasil, 2008–2010 (n = 14,375).

	Do not drink regularly	Up to 4 drinks per week	4 to 7 drinks per week	7 to 14 drinks per week	More than 14 drinks per week
	n = 7,442	n = 3,306	n = 1,365	n = 1,436	n = 826
**Sex (%)**					
Men	32.8	44.2	60.4	75.5	88.0
**Age (years)**					
Median (P25-P75)	51.0 (45.0–58.0)	51.0 (45.0–58.0)	52.0 (46.0–59.0)	52.0 (46.0–59.0)	52.0 (47.0–58.0)
**Skin color / Race** (%)					
White (n = 7,514)	48.2	58.6	59.1	53.2	50.4
Brown (‘Pardo’) (n = 4,042)	30.1	24.6	24.5	27.0	31.8
Black (n = 2,308)	17.4	13.6	13.8	17.6	15.5
Other (n = 511)	4.3	3.2	2.6	2.2	2.3
**Smoking** (%)					
Current smoker	8.9	12.9	17.0	19.6	30.8
Ex-smoker	26.6	28.3	35.5	40.7	39.8
Never smoked	64.5	58.8	47.5	39.7	29.4
**Educational level** (%)					
Incomplete elementary school	7.1	3.4	4.3	6.0	7.3
Complete elementary school	7.5	4.9	5.4	6.3	9.6
Complete secondary school	38.1	30.6	28.0	31.6	36.1
University degree	47.3	61.1	62.3	56.1	47.0
**Income (US$)**^†^					
Median (P25-P75)	691 (384–1095)	876 (518–1460)	979 (518–1460)	845 (403–1460)	691 (384–1306)
**Social class** (%)					
Low	27.3	18.8	18.2	22.7	28.3
Middle	44.8	39.3	35.5	35.4	37.4
High	26.3	40.3	44.4	40.7	32.0
Unknown	1.6	1.6	1.9	1.2	2.3
**BMI, kg/m**^**2**^ (%)					
<18.5	1.1	0.8	0.4	1.0	1.2
18.5–24.9	35.9	39.6	37.0	31.8	27.9
25–29.9	38.6	38.6	44.0	47.1	44.4
≥30	24.4	21.0	18.6	20.1	26.5
**Leisure Time Physical Activity (MET-minutes/week)**					
None (%)	48.8	37.9	32.3	35.3	39.8
Median (P25-P75) when > 0	720 (396–1440)	834 (396–1598)	834 (441–1611)	960 (480–1638)	876 (396–1590)
**Alcohol consumption with meals** (%)					
More frequently with meals	-	44.1	41.5	33.9	24.4
Both with and outside of meals	-	6.4	8.1	8.8	10.9
More frequently outside of meals	-	49.5	50.4	57.3	64.7
**Predominant beverage** (%)					
Wine	-	34.4	33.7	23.0	13.0
Beer	-	52.9	53.5	59.8	73.2
Other	-	12.7	12.8	17.2	13.8
**Metabolic syndrome** (%)	43.9	39.6	40.4	50.1	58.2
**Elevated blood pressure** (%)	43.4	41.2	41.3	50.9	59.4
**Elevated fasting glucose** (%)	69.0	68.5	75.3	82.0	84.3
**Elevated triglycerides** (%)	28.1	29.2	31.2	41.8	51.3
**Reduced HDL-C** (%)	22.9	15.6	12.8	11.6	10.3
**Elevated waist circumference** (%)	64.9	59.4	60.9	60.7	65.6

P25-75 = Percentile 25–75

^†^ Net monthly income *per capita;* 2009 conversion rate of 1.8 Brazilian reais = 1 US dollar.

Table 2 shows the same characteristics of the participants by quantity of alcohol consumed, presented separately for consumption mostly with meals or mostly outside of meals. The unadjusted prevalence of the metabolic syndrome was always greater with consumption outside of meals. S1 Table presents these characteristics by the predominant type of beverage consumed (wine or beer). The unadjusted prevalence of the metabolic syndrome was notably greater with beer consumption, independently of quantity consumed.

**Table 2:** Characteristics of participants, by timing of alcohol consumption (mostly with/outside of meals). ELSA-Brasil, 2008–2010 (n = 14,375).

	Do not drink regularly	Up to 4 drinks per week		4 to 7 drinks per week		7 to 14 drinks per week		More than 14 drinks per week	
		With	Outside	With	Outside	With	Outside	With	Outside
	n = 7,442	n = 1,459	n = 1,635	n = 566	n = 689	n = 487	n = 823	n = 202	n = 534
**Sex (%)**									
Men	32.8	42.9	46.4	59.9	61.8	76.4	75.7	90.6	87.8
**Age (years)**									
Median (P25-P75)	51 (45–58)	53 (46–59)	49 (44–56)	55 (47–62)	50 (45–56)	56 (48–62)	50 (45–56)	55 (48–62)	51 (46–57)
**Skin color / Race (%)**									
White (n = 7,514)	48.2	68.1	49.3	71.4	48.8	70.2	41.6	68.3	42.3
Brown (‘Pardo’) (n = 4,042)	30.1	18.8	30.5	18.4	29.3	15.0	35.0	18.8	37.6
Black (n = 2,308)	17.4	9.9	16.9	7.8	18.7	12.5	21.0	11.9	17.0
Other (n = 511)	4.3	3,2	3.3	2.4	3.2	2.3	2.4	1.0	3.1
**Smoking** (%)									
Current smoker	8.9	8.0	17.5	9.7	24.0	10.9	24.0	15.9	36.9
Ex-smoker	26.6	26.9	28.9	36.2	35.1	44.1	38.8	48.5	35.4
Never smoked	64.5	65.1	53.6	54.1	40.9	45.0	37.2	35.6	27.7
**Educational level** (%)									
Incomplete elementary school	7.1	2.1	4.5	3.2	5.2	4.3	7.4	5.0	8.6
Complete elementary school	7.5	3.7	6.3	3.4	7.7	5.5	7.0	6.9	11.4
Complete secondary school	38.1	22.5	39.0	17.0	37.6	19.9	40.5	22.8	42.5
Universuty degree	47.3	71.7	50.2	76.4	49.5	70.3	45.1	65.3	37.5
**Income (US$)**^†^									
Median (P25-P75)	691 (384–1095)	1095 (633–1498)	691 (384–1152)	1095 (806–1729)	691 (384–1152)	1095 (633–1498)	691 (384–1095)	1095 (576–1460)	576 (345–1095)
**Social class** (%)									
Low	27.3	13.9	24.0	13.8	23.1	18.5	26.7	20.3	33.2
Middle	44.8	32.2	46.5	22.8	45.1	21.4	44.1	22.8	42.7
High	26.3	52.4	28.0	60.3	30.9	58.3	28.3	54.5	22.1
Unknown	1.6	1.5	1.5	3.1	0.9	1.8	0.9	2.4	2.0
**BMI, kg/m**^**2**^ (%)									
<18.5	1.1	1.0	0.6	0.5	0.4	0.6	1.2	0.5	1.7
18.5–24.9	35.9	42.7	37.2	37.6	36.1	33.9	30.7	27.2	27.7
25–29.9	38.6	36.5	40.3	44.3	43.1	48.9	45.6	48.0	42.1
≥30	24.4	19.8	21.9	17.6	20.4	16.6	22.5	24.3	28.5
**Leisure Time Physical Activity (MET-minutes/week)**									
if = 0 (%)	48.8	32.4	42.6	25.3	38.2	25.7	41.7	24.3	45.5
Median (P25-P75) when >0	720 (396–1440)	800 (396–1554)	875 (400–1680)	819 (419–1588)	819 (452–1611)	960 (480–1671)	960 (480–1592)	924 (438–1676)	792 (396–1470)
**Predominant beverage** (%)									
Wine	-	49.9	20.0	53.0	18.9	46.6	8.3	31.2	4.3
Beer	-	36.9	67.9	32.0	69.5	32.9	77.0	50.0	83.9
Other	-	13.2	12.1	15.0	11.6	20.5	14.7	18.8	11.8
**Metabolic syndrome** (%)	43.9	37.1	41.7	37.3	43.3	47.0	51.8	53.5	59.7
**Elevated blood pressure** (%)	43.4	40.6	42.1	40.5	42.5	48.9	52.7	54.5	61.4
**Elevated fasting glucose** (%)	69.0	67.8	69.6	75.8	75.8	81.3	81.8	85.2	83.5
**Elevated triglycerides** (%)	28.1	25.8	32.1	26.3	35.0	34.7	45.9	42.1	54.9
**Reduced HDL-C** (%)	22.9	14.3	16.6	10.3	14.7	8.8	12.3	10.4	10.3
**Elevated waist circumference** (%)	64.9	57.4	60.3	59.7	61.7	60.4	60.6	63.4	66.1

P25-75 = Percentile 25–75

^†^ Net monthly household income *per capita;* 2009 conversion rate of 1.8 Brazilian reais = 1 US dollar.

**Fig 1:**
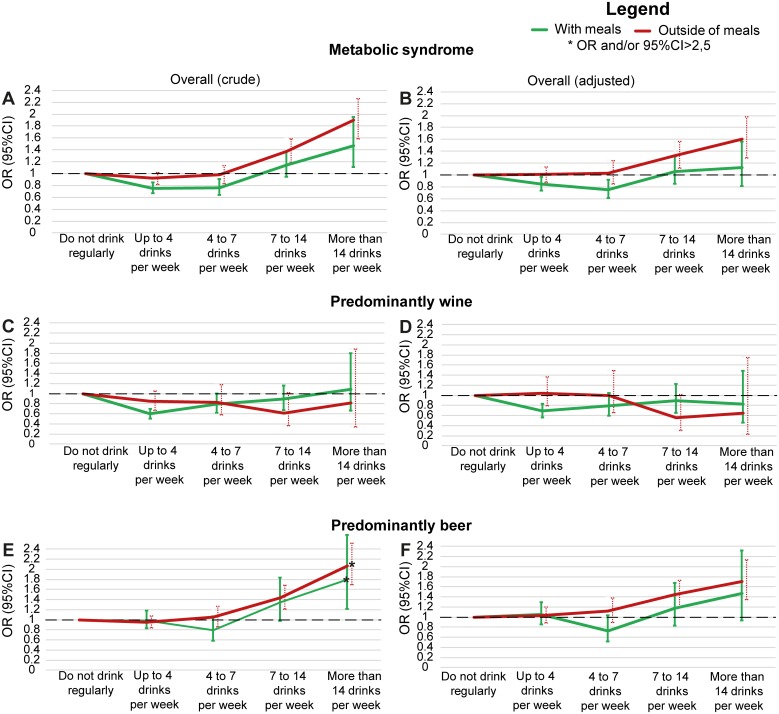
Association between alcohol consumption and the metabolic syndrome, when this consumption was either mostly with or mostly outside of meals. (A) Crude. (B) Adjusted through logistic regression for sex, age, skin color/race, smoking, BMI, educational level, net monthly household income *per capita*, social class and physical activity. (C and E) Crude, additionally stratified by predominant consumption of wine or beer. (D and F) Adjusted for the same covariates, and additionally stratified by predominant consumption of wine or beer.

Fig 1A and 1B presents the crude and adjusted associations of the metabolic syndrome with the quantity of alcohol consumed, respectively, when stratified by consumption mostly with or mostly outside of meals. Since we observed no difference in the alcohol—metabolic syndrome association between men and women (p = 0.18), we adjusted models for sex, but did not include sex interaction terms in analyses. In adjusted analysis, the consumption of alcohol most frequently with meals, when compared to abstention/occasional drinking, was inversely associated with the metabolic syndrome, OR = 0.85 (95%CI 0.74–0.97) for up to 4 drinks/week and OR = 0.75 (95%CI 0.61–0.92) for 4–7 drinks/week. In contrast, alcohol consumption taken most frequently outside of meals and in greater quantity was associated with an increased odds of the metabolic syndrome OR = 1.32 (95%CI 1.11–1.57) for 7–14 drinks/week and OR = 1.60 (95%CI 1.29–1.98) for >14 drinks/week (p for overall interaction with timing = 0.01). Greater quantities with meals and lesser quantities outside of meals showed no statistically significant associations.

**Fig 2:**
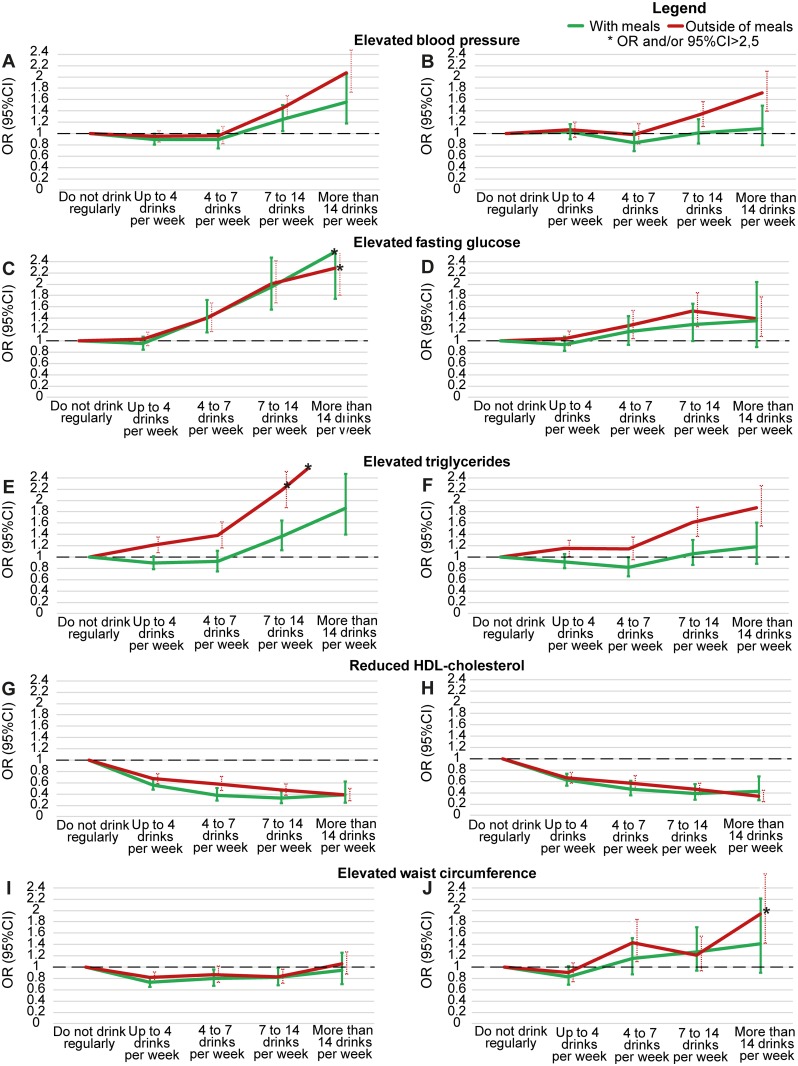
Association between alcohol consumption and components of the metabolic syndrome, when this consumption was either mostly with or mostly outside of meals. (A, C, E, G and I) Crude. (B, D, F, H and J) Adjusted through logistic regression for sex, age, skin color/race, smoking, BMI, educational level, net monthly household income *per capita*, social class and physical activity.

Fig 2 presents crude and adjusted associations of alcohol consumption with metabolic syndrome components according to the timing of such consumption. Taking as a reference abstention/occasional drinking, in the adjusted analyses lesser (<7 drinks/week) consumption of alcohol, when mostly taken with meals, was inversely associated, in at least one category of quantity consumed, with elevated triglycerides and low HDL-C. In contrast, >7 drinks/week when mostly taken outside of meals was associated, in at least one category of quantity consumed, with an increased odds of elevated blood pressure, elevated fasting glucose, elevated triglycerides and elevated waist circumference. Consumption both with and outside of meals was inversely associated with low HDL-C at all levels of consumption.

The predominance of wine or beer modified the above associations for the metabolic syndrome (p = 0.002), and for its components elevated blood pressure (p = 0.001) and hypertriglyceridemia (p = 0.009). Thus, we further stratified analyses with crude results for the metabolic syndrome being presented in Fig 1C (for wine) and Fig 1E (for beer) and equivalent adjusted analyses in Fig 1D and 1F. In comparison with the results of Fig 1B, predominantly wine drinkers (Fig 1D) did not display an increasing association with the metabolic syndrome as quantity increased, with the only statistically significant association being seen for 1–4 drinks/week mostly with meals (OR = 0.69, 95%CI 0.56–0.83). Predominantly beer drinkers (Fig 1F), on the other hand, showed an increasing association with the metabolic syndrome at greater quantities ingested, being monotonically greater and statistically significant only at higher quantities consumed mostly outside of meals (OR = 1.45, 95%CI 1.20–1.76 for 7–14 drinks/week; and OR = 1.70, 95%CI 1.35–2.14 for more than 14 drinks/week).

**Fig 3:**
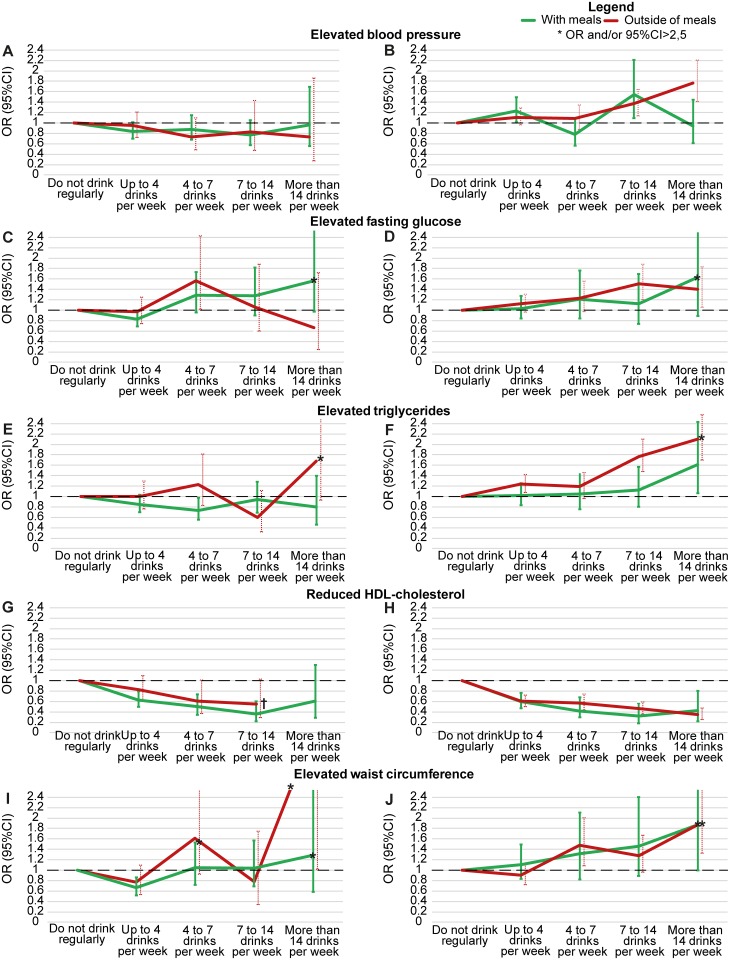
Association between alcohol consumption and components of the metabolic syndrome, when this consumption was either mostly with or mostly outside of meals. Data are presented separately by predominant beverage type. Adjusted associations. Adjustment performed through logistic regression for sex, age, skin color/race, smoking, BMI, educational level, net monthly household income *per capita*, social class and physical activity. (A, C, E, G and I) Predominantly wine. (B, D, F, H and J) Predominantly beer.

Fig 3 presents the adjusted associations of quantity of alcohol consumed with individual syndrome components separately for both timing and for the predominant beverage consumed, wine or beer. Equivalent crude associations are shown in S1 Fig. With respect to elevated blood pressure, predominantly wine consumption, independent of its timing, tended to associate with less elevated blood pressure, whereas predominantly beer consumption, when taken outside of meals in greater quantity, was associated with a monotonically increasing greater frequency of elevated blood pressure. For elevated fasting glucose, quantities of predominantly wine consumption showed no consistent pattern, whereas predominantly beer consumption outside of meals was consistently associated with greater frequencies. For elevated triglycerides, lesser quantities of consumption, when predominantly wine, were marginally associated with lesser frequencies, whereas greater quantities of consumption, when predominantly beer, were associated with greater frequencies, especially outside of meals. Greater alcohol consumption was always associated with lesser frequencies of low HDL-C. Finally, for elevated waist circumference, an inconsistent association was seen for predominantly wine drinkers, whereas greater consumption by predominantly beer drinkers was associated with greater waist circumference, independent of timing. Throughout these comparisons, consumption of wine in lesser quantities with meals was generally more protective than when taken outside of meals, although confidence intervals almost always overlapped. No clear pattern was seen with respect to timing of consumption for predominantly beer drinkers at greater quantities, with exception of triglycerides (p = 0.04 for overall heterogeneity across timings).

Analyses adjusting additionally for binge drinking and for a previous diagnosis and/or drug treatment for diabetes, hypertension and dyslipidemia produced no important differences in results (data not shown).

## Discussion

In this large sample, the association of alcohol ingestion with the metabolic syndrome depends on much more than the mere quantity ingested. When taken with meals, alcohol was consistently associated with a lower frequency of the metabolic syndrome than when taken outside of meals. This translated into the syndrome being significantly less frequent in those taking alcohol in smaller quantities with meals, and significantly more frequent in those taking alcohol in larger quantities outside of meals. Beverage type appeared to explain much, but not all, of these associations, with wine in small doses being generally associated with lower frequency of the syndrome and its components, and beer, in larger doses, being associated with a greater frequency of the syndrome and all of its components except HDL-C. Largest differences between those predominantly consuming beer or wine were seen for elevated blood pressure and elevated triglycerides. Consistent with many previous studies, greater alcohol consumption, independent of timing or beverage type, was associated with higher HDL-C levels [13, 21, 22, 24, 35, 36]. Most alcohol was consumed in metabolically unfavorable type and timing.

A recent meta-analysis summarized data on the association of quantity of alcohol consumed with the metabolic syndrome [37]. In comparison with non-drinkers in this meta-analysis, light consumption of alcoholic beverages (≤5 g/day) was associated with a lower risk of the syndrome (RR = 0.86, 95%CI 0.75–0.99), while excessive consumption (>35 g/day) was associated with greater risk (RR = 1.84, 95%CI 1.34–2.52). However, we know of no previous study which has incorporated quantity, timing and beverage type in the investigation of alcohol consumption´s associations with the metabolic syndrome.

The few studies which have investigated the influence of timing of consumption with respect to meals on metabolic syndrome components report findings in consonance with ours. The prevalence of elevated blood pressure among non-drinking Italians (42.8%) and Americans (40.4%) was higher than among those who drank alcohol with meals (36.6% and 39.8%), but not higher than among those who drank alcohol outside of meals (42.8% and 41.4%) [7].

Few studies have investigated major clinical endpoints. An Italian case-control study found that, in comparison with non-drinkers, odds of having a myocardial infarction were 50% lower (OR = 0.50, 95%CI 0.30–0.82) for those taking 3 or more drinks/day with meals, but not among those who consumed a similar quantity outside of meals (OR = 0.98, 95%CI 0.49–1.96) [15]. In an additional large cohort study, in comparison with non-drinkers, men who drank wine with meals, but not those who drank outside of meals, had lower risk of death from all causes (RR = 0.64, 95%CI 0.49–0.82), from cardiovascular diseases (RR = 0.50 95%CI 0.33–0.74); and from coronary heart disease (RR = 0.48 95%CI 0.30–0.76). Among women in this study, for whom many fewer deaths accrued, those who drank wine outside of meals experienced much greater all-cause mortality (RR = 3.48, 95%CI 1.25–9.66). Independent of the quantity of alcohol consumed, those who drank with meals experienced significantly lower all-cause death rates than those who drank outside of meals [14].

Although a greater number of studies exist, the literature is less clear on the relative effects of different types of beverages on cardiometabolic disease. A meta-analysis evaluating the relationship between beer, wine and distillates with cardiovascular disease found similar dose-response curves for beer and wine, both showing a reduction of approximately 33% in events for those who consumed 25 g/d [11]. One cross-sectional study reported 40% greater odds (95%CI 10%– 70%) of having a large waist-hip ratio in non-wine (predominantly beer) drinkers, and a 50% lesser odds (95%CI 21%—95%) in wine drinkers [38]. A meta-analysis suggested that beer consumption, especially in larger quantities, is associated with large waist circumference [39]. Sun and colleagues [37], in a recent meta-analysis of alcohol consumption and the metabolic syndrome, found an insufficient number of studies to evaluate differences in risk of the syndrome by beverage type.

The apparent cardioprotective effects of alcohol ingestion may act through a series of mechanisms, including anti-inflammatory effects, inhibition of platelet aggregation and alterations in lipid metabolism [40]. Those who drink moderate quantities of alcohol had lower levels of several inflammation markers, including fibrinogen, C-reactive protein (CRP), leucocytes and elevated plasma viscosity [41]. Experimental studies in isolated adipose tissue show that alcohol decreases levels of interleukin-6 (IL-6), interleukin-8 (IL-8), tumor necrosis factor-α (TNF-α), and monocyte chemotaxic factor -1 (MCP-1) [42].

An anti-inflammatory effect of alcohol with meals might provide greater benefit than one occurring outside of meals. Nutrient overload produces oxidative stress [43]. A diet rich in corn oil and lard stimulates greater absorption of lipopolysaccharide, a major pro-inflammatory stimulant, from the gut [44]. In a small randomized trial, consumption of 4 drinks of wine with dinner for 4 weeks increased total plasma anti-oxidant capacity and suppressed nuclear factor kappa B (NF-κB) activation in peripheral blood mononuclear cells 1 hour after a meal [45].

Alcohol has also been shown, in vitro, to lower endothelial adhesion molecule expression [46] and, in moderate doses, to increase the release of nitric oxide from the endothelium [47]. Moderate consumption also increases HDL-C and alters many of its actions as well as inhibits oxidation of LDL-C and other apoB-containing lipoproteins [48]. A small, short-term clinical trial demonstrated that red wine taken with a mid-day meal reduced post-prandial blood pressure and nocturnal dipping in systolic blood pressure [49]. Another small randomized trial demonstrated that alcohol taken with white bread significantly lowered post-prandial hyperglycemia [50].

On other hand, our study highlights that among the alcohol consumers in this sample of Brazilians, many drank heavily, the majority drank alcohol most frequently outside of meals, and most were predominantly beer drinkers. Even those who drank mostly with meals, less than half drank mainly wine. Thus, the main patterns of alcohol consumption in this study are all associated with a worse cardio-metabolic risk profile. Other studies also demonstrate a higher risk for cardio-metabolic abnormalities associated with excessive alcohol intake. A Korean epidemiologic study shows that heavy alcohol consumption was associated with significantly higher odds ratios for high blood pressure (30–79.9g/d: OR = 1.45, 95%CI 1.12–1.87; ≥80g/d: OR = 1.88, 95%CI 1.32–2.68), high triacylglycerol (30–79.9g/d: OR = 1.37, 95%CI 1.05–1.77; ≥80g/d: OR = 1.76, 95%CI 1.23–2.50) and large waist (≥80g/d: OR = 2.02, 95%CI 1.22–3.34) in men, and high fasting blood glucose (≥30g/d: OR = 2.12, 95%CI 1.13–3.97) and high triacylglycerol (≥30g/d: OR = 2.19, 95%CI 1.21–3.97) in women. Odds ratios for the metabolic syndrome and its components tended to increase with increasing alcohol consumption [51]. Kim and colleagues [52] also found, comparing with non-drinkers, heavy drinkers (≥30g/d) suffered the largest incidence of metabolic syndrome (OR = 2.11; 95%CI 1.25–3.56). In a Mediterranean cohort [22], people who consumed ≥7drinks/week presented a significantly higher risk of developing metabolic syndrome (OR = 1.80; 95%CI 1.22–2.66), hypertriglyceridemia (OR = 2.07; 95%CI 1.46–2.93) and impaired fasting glucose (OR = 1.54; 95%CI 1.16–2.04) when compared with non-drinkers. In addition, beer consumption was associated with higher risk for the metabolic syndrome (p for trend = 0.03) and a higher risk of hypertriglyceridemia (OR = 1.81; 95%CI 1.02–3.20).

Furthermore, the harmful use of alcohol contributes to many other types of disease and injury. Worldwide, harmful alcohol consumption is a component cause of more than 200 disease and injury conditions, and is believed responsible for 5.9% of all deaths and 5.1% of overall disability, most notably those due to liver cirrhosis, cancers and injuries, as well as due directly to alcohol dependence [6]. Additionally, alcohol consumption contributes in large measure to the excessive violence that permeates Brazilian society [53].

National guidelines for alcohol drinking frequently discourage consumption [54]. The 2015–2020 Dietary Guidelines for Americans recommend that if alcohol is consumed, it should be in moderation, and explicitly do not recommend that abstainers begin drinking for any reason [55]. Yet alcohol consumption remains persistent in societies across the world, despite the huge disease burden it produces. Our findings suggest that much of the untoward cardio-metabolic effects of alcohol consumption may be due to habits other than mere quantity consumed, which, while dominant in the ELSA-Brasil sample, are logically susceptible to change. Two recent guidelines, from Canada and Australia [56, 57], emphasize that alcohol is best consumed with meals. However, their justification is based on the observation that meals slow the absorption of alcohol, rather than that such timing of consumption is associated with a more favorable cardiometabolic profile.

Important strengths of our study are its large sample size, standardized measurement techniques, and the inclusion of questions which permit the more comprehensive analysis presented. However, limitations to our report also merit comment. The cross-sectional nature of our analyses make inferences of causality more difficult. Self-report of alcohol consumption is subject to recall bias, especially, as in our case, when assessment is obtained by interview, as participants tend to give answers perceived as socially desirable. Due to this bias, alcohol consumption might be underestimated, especially in groups with higher consumption. It is unclear, however, how disease presence would bias associations with timing of consumption and beverage type, the more novel and relevant aspects of our findings. Additionally, residual confounding, particularly by socioeconomic status, must always be considered, given that alcohol consumption is a very socially determined habit.

## Conclusions

In conclusion, we have found that the association of alcohol consumption with the metabolic syndrome and its components differs by the timing of such consumption and beverage type. Specifically, light consumption of alcoholic beverages with meals was associated with a lesser frequency of the syndrome and many of its elements, while greater consumption, particularly outside of meals, was associated with higher frequencies. Wine, which was taken mainly with meals, appeared to explain part of the mealtime associations, while beer, taken mainly outside of meals, appeared to explain part of associations of consumption outside of meals. If further investigations confirm these findings, and demonstrate that similar associations exist for clinical endpoints such as myocardial infarction and diabetes, public policies, in addition to favoring interventions to limit harmful consumption, should recommend that alcohol, when taken, should be preferably consumed with meals.

## Supporting Information

S1 Fig Alcohol consumed, mostly with/outside of meals, predominant wine or beer, and components of metabolic syndrome. Crude association. Left side panels A, C, E, G and I: predominant wine. Right side panels B, D, F, H and J: predominant beer.
(TIF)

S1 Table Characteristics of participants, by type of beverage predominantly consumed (mostly wine/beer). ELSA-Brasil, 2008–2010 (n = 5,978). P25-75 = Percentile 25–75 ^†^Net monthly income *per capita;* 2009 conversion rate of 1.8 Brazilian reais = 1 US dollar.
(DOCX)
