# Supplementary figures and images for: Timing and Type of Alcohol Consumption and the Metabolic Syndrome - ELSA-Brasil
Source: PLoS One. 2016 Sep 19;11(9):e0163044. doi: 10.1371/journal.pone.0163044 (PMC5028065; doi:10.1371/journal.pone.0163044)

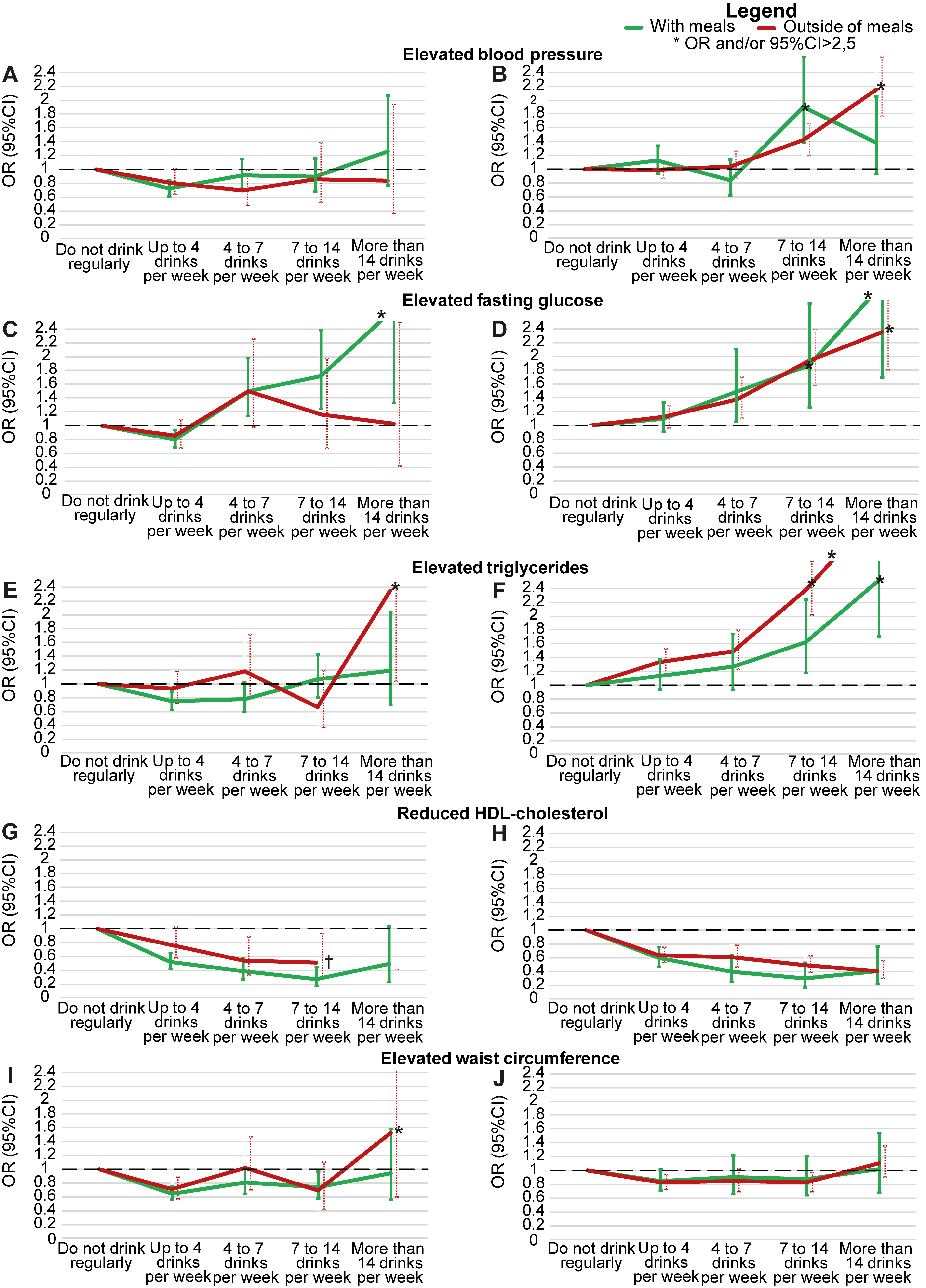

Supplement: S1 Fig — Crude association. Left side panels A, C, E, G and I: predominant wine. Right side panels B, D, F, H and J: predominant beer. (TIF) [file pone.0163044.s001.tif]
