# Supplementary material for: Timing and Type of Alcohol Consumption and the Metabolic Syndrome - ELSA-Brasil
Source: PLoS One. 2016 Sep 19;11(9):e0163044. doi: 10.1371/journal.pone.0163044 (PMC5028065; doi:10.1371/journal.pone.0163044)
Supplement: S1 Table — P25-75 = Percentile 25–75 †Net monthly income per capita; 2009 conversion rate of 1.8 Brazilian reais = 1 US dollar. (DOCX) [file pone.0163044.s002.docx]

S1 Table. Characteristics of participants, by type of beverage predominantly consumed (mostly wine/beer). ELSA-Brasil, 2008-2010 (n=5,978).

|  | | **Up to 4 drinks**  **per week** | | **4 to 7 drinks**  **per week** | | **7 to 14 drinks**  **per week** | | **More than 14 drinks**  **per week** | |
| --- | --- | --- | --- | --- | --- | --- | --- | --- | --- |
|  | | **Wine** (n=1,138) | **Beer** (n=1,748) | **Wine**  (n=460) | **Beer**  (n=731) | **Wine**  (n=330) | **Beer**  (n=859) | **Wine**  (n=107) | **Beer**  (n=605) |
| **Sex (%)** | |  |  |  |  |  |  |  |  |
|  | Men | 35.3 | 48.1 | 50.9 | 64.7 | 63.0 | 77.4 | 80.4 | 89.3 |
| **Age (years)** | |  |  |  |  |  |  |  |  |
|  | Median (P25-P75) | 53.0  (46.0-60.0) | 49.0  (44.0-56.0) | 55.0  (48.0-62.0) | 50.0  (44.0-56.0) | 56.0  (48.0-62.0) | 50.0  (45.0-56.0) | 57.0  (49.0-64.0) | 51.0  (46.0-57.0) |
| **Skin color / Race (%)** | |  |  |  |  |  |  |  |  |
|  | White | 67.0 | 51.9 | 75.9 | 47.1 | 75.2 | 42.3 | 82.2 | 43.3 |
|  | Brown (‘Pardos’) | 21.2 | 26.7 | 14.6 | 30.6 | 15.7 | 32.5 | 11.2 | 35.5 |
|  | Black | 8.8 | 17.9 | 7.6 | 19.6 | 7.6 | 22.7 | 4.7 | 19.0 |
|  | Other | 3.0 | 3.5 | 1.9 | 2.7 | 1.5 | 2.5 | 1.9 | 2.2 |
| **Smoking** (%) | |  |  |  |  |  |  |  |  |
|  | Current smoker | 6.6 | 16.3 | 9.8 | 21.5 | 6.9 | 23.4 | 14.0 | 33.1 |
|  | Ex-smoker | 27.5 | 29.3 | 34.3 | 34.9 | 45.5 | 38.6 | 46.7 | 38.3 |
|  | Never smoked | 65.9 | 54.4 | 55.9 | 43.6 | 47.6 | 38.0 | 39.3 | 28.6 |
| **Educational level** (%) | |  |  |  |  |  |  |  |  |
|  | Incomplete elementary school | 1.6 | 4.2 | 1.5 | 6.2 | 0.9 | 7.3 | 1.9 | 8.3 |
|  | Complete elementary school | 3.5 | 6.4 | 1.3 | 8.1 | 3.6 | 7.7 | 2.8 | 11.1 |
|  | Complete secondary school | 20.5 | 39.3 | 13.5 | 39.1 | 12.7 | 41.9 | 11.2 | 41.5 |
|  | Universuty degree | 74.4 | 50.1 | 83.7 | 46.6 | 82.7 | 43.1 | 84.1 | 39.1 |
| **Income (US$)^†^** | |  |  |  |  |  |  |  |  |
|  | Median (P25-P75) | 1095  (691-1613) | 691  (393-1152) | 1152  (864-2074) | 691  (384-1152) | 1152  (864-2190) | 633  (384-1095) | 1460  (1037-2190) | 599  (345-1095) |
| **Social class** (%) | |  |  |  |  |  |  |  |  |
|  | High | 53.5 | 29.2 | 65.0 | 28.9 | 70.0 | 26.9 | 72.0 | 23.8 |
|  | Middle | 32.0 | 45.8 | 24.6 | 44.2 | 18.2 | 44.4 | 15.9 | 43.0 |
|  | Low | 13.0 | 23.3 | 7.8 | 25.6 | 9.7 | 27.9 | 8.4 | 31.4 |
|  | Unknown | 1.5 | 1.7 | 2.6 | 1.3 | 2.1 | 0.8 | 3.7 | 1.8 |

S1 Table. Characteristics of participants, by type of beverage predominantly consumed (mostly wine/beer). ELSA-Brasil, 2008-2010 (n=5,978) (continued).

|  | | **Up to 4 drinks**  **per week** | | **4 to 7 drinks**  **per week** | | **7 to 14 drinks**  **per week** | | **More than 14 drinks**  **per week** | |
| --- | --- | --- | --- | --- | --- | --- | --- | --- | --- |
|  | | **Wine** (n=1,138) | **Beer** (n=1,748) | **Wine**  (n=460) | **Beer**  (n=731) | **Wine**  (n=330) | **Beer**  (n=859) | **Wine**  (n=107) | **Beer**  (n=605) |
| **BMI, kg/m^2^** (%) | |  |  |  |  |  |  |  |  |
|  | <18.5 | 1.0 | 0.6 | 0.4 | 0.5 | 0.9 | 0.9 | 0.0 | 1.3 |
|  | 18.5-24.9 | 44.5 | 37.0 | 40.7 | 34.9 | 37.9 | 29.5 | 31.8 | 26.1 |
|  | 25-29.9 | 36.6 | 39.3 | 42.2 | 44.3 | 46.1 | 47.7 | 48.6 | 44.3 |
|  | ≥30 | 17.9 | 23.1 | 16.7 | 20.3 | 15.2 | 21.9 | 19.6 | 28.3 |
| **Leisure Time Physical Activity (MET-minutes/week)** | |  |  |  |  |  |  |  |  |
|  | if = 0 (%) | 31.0 | 44.0 | 19.6 | 41.7 | 24.5 | 40.7 | 17.8 | 45.5 |
|  | if > 0 Median (P25-P75) | 876  (462-1554) | 792  (396-1668) | 876  (480-1704) | 829  (396-1556) | 1032  (558-1800) | 960  (480-1638) | 849  (480-1655) | 844  (396-1500) |
| **Alcohol consumption with meals** (%) | |  |  |  |  |  |  |  |  |
|  | More frequently with meals | 64.0 | 30.8 | 65.2 | 24.8 | 68.8 | 18.6 | 58.9 | 16.7 |
|  | Both with and without meals | 7.3 | 5.7 | 6.5 | 9.7 | 10.6 | 7.6 | 19.6 | 9.3 |
|  | More frequently without meals | 28.7 | 63.5 | 28.3 | 65.5 | 20.6 | 73.8 | 21.5 | 74.0 |
| **Metabolic syndrome** (%) | | 34.4 | 42.8 | 37.4 | 43.6 | 39.1 | 53.3 | 45.8 | 61.5 |
|  |  |  |  |  |  |  |  |  |  |
| **Elevated blood pressure** (%) | | 36.0 | 44.1 | 38.3 | 43.0 | 39.7 | 53.2 | 47.7 | 60.3 |
|  |  |  |  |  |  |  |  |  |  |
| **Elevated fasting glucose** (%) | | 64.5 | 70.8 | 75.4 | 76.1 | 78.5 | 81.6 | 82.2 | 84.8 |
|  |  |  |  |  |  |  |  |  |  |
| **Elevated triglycerides** (%) | | 24.4 | 33.0 | 25.7 | 35.8 | 28.5 | 46.7 | 35.5 | 56.5 |
|  |  |  |  |  |  |  |  |  |  |
| **Reduced HDL-C** (%) | | 15.4 | 15.7 | 11.1 | 14.5 | 10.6 | 12.6 | 11.2 | 10.9 |
|  |  |  |  |  |  |  |  |  |  |
| **Elevated waist circumference** (%) | | 56.1 | 61.1 | 61.3 | 61.4 | 57.3 | 61.4 | 65.4 | 66.9 |
|  |  |  |  |  |  |  |  |  |  |

P25-75=Percentile 25-75

^†^Net monthly income *per capita;* 2009 conversion rate of 1.8 Brazilian reais = 1 US dollar
